# The maize *fused leaves1* (*fdl1*) gene controls organ separation in the embryo and seedling shoot and promotes coleoptile opening

**DOI:** 10.1093/jxb/erv278

**Published:** 2015-06-20

**Authors:** Nicoletta La Rocca, Priscilla S. Manzotti, Marina Cavaiuolo, Alessandra Barbante, Francesca Dalla Vecchia, Damiano Gabotti, Ghislaine Gendrot, David S. Horner, Jelena Krstajic, Martina Persico, Nicoletta Rascio, Peter Rogowsky, Alessio Scarafoni, Gabriella Consonni

**Affiliations:** ^1^Dipartimento di Biologia, Università degli Studi di Padova, Via Ugo Bassi 58/B, 35131 Padova, Italy; ^2^Dipartimento di Scienze Agrarie e Ambientali (DISAA), Produzione, Territorio, Energia Università degli Studi di Milano, Via Celoria 2, 20133 Milan, Italy; ^3^Université de Lyon, ENS de Lyon, INRA, CNRS, Université Lyon 1, Unité Reproduction et Développement des Plantes, F-69364 Lyon, France; ^4^Dipartimento di Bioscienze, Università degli Studi di Milano, Via Celoria 26, 20133 Milan,Italy; ^5^Dipartimento di Scienze per gli Alimenti la Nutrizione, l’Ambiente, Università degli Studi di Milano, Via Celoria 2, 20133 Milan, Italy

**Keywords:** Cuticle, embryogenesis, epicuticular waxes, *fused leaves1*, shoot development, *ZmMYB94*.

## Abstract

The *fdl1-1* mutation, caused by an *Enhancer*/*Suppressor mutator* (*En*/*Spm*) element insertion located in the third exon of the gene, identifies a novel gene encoding ZmMYB94, a transcription factor of the R2R3-MYB subfamily. The *fdl1* gene was isolated through co-segregation analysis, whereas proof of gene identity was obtained using an RNAi strategy that conferred less severe, but clearly recognizable specific mutant traits on seedlings.

*Fdl1* is involved in the regulation of cuticle deposition in young seedlings as well as in the establishment of a regular pattern of epicuticular wax deposition on the epidermis of young leaves. Lack of *Fdl1* action also correlates with developmental defects, such as delayed germination and seedling growth, abnormal coleoptile opening and presence of curly leaves showing areas of fusion between the coleoptile and the first leaf or between the first and the second leaf. The expression profile of *ZmMYB94* mRNA—determined by quantitative RT-PCR—overlaps the pattern of mutant phenotypic expression and is confined to a narrow developmental window. High expression was observed in the embryo, in the seedling coleoptile and in the first two leaves, whereas RNA level, as well as phenotypic defects, decreases at the third leaf stage. Interestingly several of the *Arabidopsis MYB* genes most closely related to *ZmMYB94* are also involved in the activation of cuticular wax biosynthesis, suggesting deep conservation of regulatory processes related to cuticular wax deposition between monocots and dicots.

## Highlight

This study provides the first characterization of an R2R3 family MYB transcription factor involved in cuticle and epicuticular wax deposition, whose action is confined to maize embryogenesis and juvenile phase.

## Introduction

Seedling architecture is established during maize embryogenesis, leading to the formation of a well developed embryo axis consisting of the embryonic primary root and the embryonic shoot separated by the scutellar node and encapsulated by two protective structures, the coleoriza for the root and the coleoptile for the shoot. The shoot includes the first internode, called the mesocotyl, located between the scutellar node and the coleoptilar node, and—depending on the genetic background—five or six short internodes located above the coleoptilar node, with a leaf primordium at each node. Each leaf primordium encircles those below, thus forming a conical structure that is encapsulated in the coleoptile, which develops as a sheathing structure enveloping the stem tip and the embryonic leaves (Abbe and Stein, 1954). Coleoptile and leaf primordia, which are initiated from 8 days after pollination (DAP) onwards (Clark, 1996), derive from distinct origins, the former arising as a ring on the surface of the scutellum and the latter from the shoot apical meristem (SAM) (Abbe and Stein, 1954; Bommert and Werr, 2001). The coleoptile is the first organ to grow when seed germination occurs. It appears as a conical structure that elongates and pierces the soil, enclosing and thus protecting the young leaves in the shoot apex. In this initial phase leaf elongation keeps pace with that of the coleoptile. Later on, the coleoptile arrests its growth and opens at its apex, wherefrom leaves emerge. The coleoptile dies soon after leaf emergence.

This study describes the maize *fused leaves1-1* (*fdl1-1*) mutant, which exhibits abnormalities during embryo development and the first phases of seedling growth, between coleoptile emergence and disclosure of the third leaf. The main features of mutant plants are irregular coleoptile opening and the presence of regions of adhesion between the coleoptile and the first leaf and between the first and second leaves. In the adherent regions the cuticle is not deposited. In addition epicuticular wax deposition on the epidermis of young leaves is irregular.

MYB (myeloblastosis) family proteins possess a conserved DNA-binding domain (DBD), which is homologous to the DBD of animal c-Myb (Klempnauer *et al.*, 1982) and is composed of up to three imperfect repeats (named sequentially as R1, R2 and R3, respectively) (Dubos *et al.*, 2010). The R2R3 subclass of MYB factors contains two repeats and is the most common type in plants (Du *et al.*, 2013). 157 genes encoding R2R3-MYB proteins have been identified in the maize genome and classified into 37 subgroups, according to their structure and phylogenetic relationships (Du *et al.*, 2013). However, functional studies have been performed for only a few maize *myb* genes that are involved in the control of the phenylpropanoid metabolic pathway (Paz-Ares *et al.*, 1987; Marocco *et al.*, 1989; Grotewold *et al.*, 1991; Fornalé *et al.*, 2006; Heine *et al.*, 2007).

Here we show that the *fdl1-1* mutant phenotype is attributable to the insertion of an *Enhancer*/*Suppressor mutator* (*En*/*Spm*) element in *ZmMYB94,* an R2R3 family MYB transcription factor that appears to be part of a monocot-specific subfamily expansion. Its functional analyses indicate that during maize embryo and seedling development, cuticle deposition is required to establish correct organ morphogenesis and the formation of boundaries between organs, while phylogenetic reconstructions suggest conservation of roles for MYB94-like proteins in the regulation of wax deposition between monocots and dicots.

## Materials and methods

### Mutant isolation and propagation

The *fdl1-1* mutation, previously designated as *fdl347*, was first identified by Professor Jane A. Langdale (Department of Plant Science, University of Oxford, Oxford, UK) in the selfed progeny of a maize (*Zea mays*) stock crossed as female to an *En*/*Spm* line. The F_2_ progeny segregated for normal and defective seedlings in a 3:1 ratio, showing that *fdl1-1* behaves as a monogenic recessive mutation. The *fdl1-1* allele was maintained in homozygosis as well as in heterozygosis. Heterozygous plants were obtained by outcrossing plants homozygous for the mutation to B73 and H99 maize inbred lines.

Wild-type and mutant maize seeds were germinated in a growth chamber at 25°C on wet filter paper. Seeds were kept in the dark for 4–5 d and then transferred to a 14h light photoperiod and photon fluence of 70 µmol m^-2^ s^-1^.

For embryo-rescue experiments, 12 and 18 DAP immature embryos were excised and cultivated *in vitro*, as described by Dolfini *et al.*, (1999).

### *fdl1-1* mapping

Crosses of heterozygous *+/fdl1-1* females to stocks carrying the TB-A translocation (Maize Genetics Cooperation Stock Center) were used to position *fdl1* on the long arm of chromosome 7. Individuals bearing a B-A translocation produce hypoploid sperms, lacking an extended portion of a specific chromosome arm, which can be used to fertilize a female donor plant carrying the mutation under study. The recovery of hemizygous mutant embryos allows localization of a recessive gene to the chromosome arm involved (Beckett, 1978).

*fdl1-1* was then mapped in segregating F_2_ populations using simple sequence repeat (SSR) markers. F_1_ seedlings were obtained from the cross between the homozygous *fdl1-1* stock and the H99 inbred line. F_1_ plants were then self-pollinated to produce F_2_ progenies. The genomic DNA was extracted from parental and F_1_ plants, and from individual mutant and wild-type F_2_ seedlings, and was amplified with primers for SSR markers on chromosome 7L. DNA extraction, reaction conditions and gel electrophoresis were carried out as described in the SSR protocols available from the Maize Genetics and Genomic Database (http://www.maizegdb.org). Mapping data were analysed with the mapmaker/EXP3.0 software (Lander *et al.*, 1987).

### Light and electron microscopy

For transmission electron microscopy and light microscopy, immature 18 DAP embryos, germinating seedlings and young leaves from wild type and fdl1-1 mutants were fixed overnight at 4°C in 6% glutaraldehyde in 0.1M cacodylate buffer (pH 6.9) and post-fixed for 2h in 1% osmium tetroxide in the same buffer. The fixed samples were dehydrated in a series of ethyl alcohol and propylene oxide solutions and embedded in araldite.

For transmission electron microscopy, ultrathin sections (600nm thick) were cut with an ultramicrotome (Ultracut, Reichert-Jung, Leica Wetzlar, Germany), stained with uranyl acetate and lead citrate and examined with an electron microscope (TEM 300, Hitachi, Japan) operating at 75kV.

For light microscopy, thin sections (1 µm thick), cut with the same ultramicrotome, were stained with toluidine blue [1% toluidine blue 1% sodium tetraborate (1:1, v/v)] and examined with a light microscope (Ortholux, Leitz, Germany).

The fluorescence emission of unstained thin sections was tested using an epifluorescence microscope (AXIOSCOP, Zeiss, Germany) fitted with an excitation filter (BP 365/12), a chromatic beam splitter (FT 395) and a barrier filter (LP 397). Cell walls excited with UV light can exhibit blue autofluorescence due to the presence of phenolic components (Harris and Hartley, 1976).

For scanning electron microscopy, immature 18 DAP embryos and seedlings were fixed and post-fixed with the same procedure used for transmission electron microscopy and dehydrated in a graded ethyl alcohol series. They were then dried at critical point, coated with gold and palladium and examined with a scanning electron microscope (Stereoscan 260, Cambridge, UK) operating at 25kV.

For the analysis of epicuticular waxes, leaf pieces of wild-type, fdl1-1 mutant and RNAi transformed plants, were dried and processed according to La Rocca *et al.*, (2014). The specimen surfaces were examined with the same scanning electron microscope.

### Toluidine blue test

The toluidine blue test for detecting defects in the leaf cuticle (Tanaka *et al.*, 2004) was carried out on fdl1-1 mutant and wild-type seedlings from F_2_ segregating progenies. All the samples were submerged in an aqueous solution of 0.05% (w/v) toluidine blue (Sigma). After 3min the solution was removed and the samples were washed gently with distilled water to remove excess dye. Images were taken by using a digital camera.

### Co-segregation analysis and SSR mapping

For Southern analysis, maize genomic DNA was extracted from 7-day-old seedlings and leaf tissues using the urea extraction method (Chen and Dellaporta, 1994). Then 10 μg of DNA were digested with the *BamH1* endonuclease, which cuts once inside the *En/Spm* element, and separated on 0.8% agarose gels. DNA fragments were transferred to Hybond N^+^ membranes (Amersham), and membranes were subsequently probed with α-^32^P-labelled probes, as described in Gutierrez-Marcos *et al.*, (2007). Hybridization probes were as follows. The *En*/*Spm*-specific probe (pBX1), corresponding to a 457bp BanII-XbaI fragment internal to the *En/Spm* element, was isolated by restriction of the plasmid containing the cloned element (Pereira *et al.*, 1986) followed by gel purification. Probe1, whose length is 78bp, was amplified from the genomic clone containing the 2.6kb fragment co-segregating with the mutant phenotype, by using two primers (5′-ACTAGTGGATCCTCGGACG-3′; 5′-TGTAGTGCCCACCACAAGC-3′), corresponding to the end of the cloning vector and of the *En/Spm* element respectively.

For PCR analysis, 50ng of genomic DNA was subjected to 35 cycles of amplification with 1.25U of GoTaq® Flexi DNA Polymerase (Promega), 1× Colourless GoTaq®Flexi Buffer, 2.5mM MgCl_2_, 0.2mM dNTPs, 0.2 µM primers and 1M betaine. Annealing temperatures were 58–62°C (optimized for each primer pair, as listed in Supplementary Table S1) with an extension time of 60 s.

Primers used for co-segregation analysis included two primer sets specific for the mutant allele, which comprised a *En*/*Spm* primer and a gene specific primer (Consfdl2-F, Spm1-R; and Spm3-F, AW-R), and a primer set specific for the wild-type allele (ConsFdl2, AWR), spanning the region containing the *En/Spm* insertion (Supplementary Fig. S3A). PCR products were separated by agarose gel electrophoresis and visualized by ethidium bromide staining.

Simple sequence repeat (SSR) markers were recovered from the MaizeGDB database (www.maizegdb.org/probe.php).

### Genomic cloning and cDNA synthesis

The Spm-BamH1 2.6kb polymorphic restriction fragment co-segregating with the mutant phenotype was cloned from a subgenomic library prepared from size-fractionated BamH1 fragments of *fdl1-1/fdl1-1* genomic DNA in the pBluescript SK vector, according to the manufacturer’s instructions (Stratagene). Four independent clones were selected and the presence of the *En/Spm* element and DNA flanking the insertion was determined by sequencing.

For cDNA synthesis, total RNA was extracted with 1ml TRI reagent (Molecular Research Center), according to the supplier’s instructions and, after treating RNA samples with DNase I, (Invitrogen) for 30min at 25°C, cDNA was synthesized using SuperScript III First-Strand Synthesis System for RT-PCR (Invitrogen), starting from 5 µg of total RNA primed with oligo(dT).

For wild-type and mutant gene structure analysis, PCR were conducted on both genomic DNA and cDNA using gene specific primers (GSP) and *En/Spm* specific primers. Primer sequences, listed in Supplementary Table S1, were designed using the Primer3 software (http://www.ncbi.nlm.nih.gov/tools/primer-blast/).

PCR products were electrophoresed on agarose gels and purified with the Illustra GFX™ PCR DNA and Gel Band Purification Kit (GE Healthcare), according to the instructions of the manufacturer, and sequenced in both orientations at the CRIBI Biotechnology Center (BMR Genomics, University of Padova, Padova, Italy, http://www.bmr-genomics.it).

### RNAi

A 481bp *ZmMYB94* fragment was amplified from genomic DNA of genotype B73 with primers attB1-FDL-RNAi and attB2-FDL-RNAi (Supplementary Table S1) and recombined in inverted orientation and under the control of the constitutive rice *Actin* promoter into a derivative of the integrative plasmid pSB11 (Ishida *et al.*, 1996), which contained a Basta resistance cassette and a GFP cassette, to yield plasmid L1258. Transformation of genotype A188 was performed as described previously (Pouvreau *et al.*, 2011). To evaluate the molecular efficiency of the RNAi construct, *ZmMYB94* expression was assayed in the third leaf of primary transformants by quantitative RT-PCR in technical duplicate.

### Reverse transcription and quantitative reverse transcription PCR analysis

Tissue samples were from plants of the B73 inbred line and are listed in Supplementary Table S2. For each sample, total RNA was obtained from two biological replicates to assess the repeatability of the data.

cDNAs were diluted 100-fold, and 2 μl were used in a final volume of 20 μl containing 10 μl of iQ SYBR Green Supermix and 0.25 μM of each primer. Amplifications were carried out using an iCycler thermocycler equipped with the MyiQ detection system (Bio-Rad, Milano, Italy) in 96-well optical reaction plates sealed with optical tapes (Bio-Rad). The reaction conditions were as follows: 96°C for 30 s, 40 cycles 96°C for 30 s, 63°C for 30 s, 72°C for 30 s. All primers used are listed in Supplementary Table S1. Data are presented as the mean of two biological replicates and three technical replicates, with the exception of bracts, ear, anthers and silks, which are presented as the mean of three technical replicates.

Each reaction was performed in triplicate. No-template controls and not retro-transcribed samples were included in the experimental set. The absence of aspecifically-amplified products was checked with derivative melting curves, obtained by progressive heating at 0.3°C every 15 s. Raw data were collected and analysed with the iQ5 software (Bio-Rad, Milano, Italy) with the following parameters: baseline from the 2nd to the 10th cycle and threshold calculated automatically by the software for every reaction. Differences in gene expression were calculated by the comparative delta–delta CT method (Schmittgen and Livak, 2008) with a dedicated Microsoft Excel macro created by Bio-Rad. All quantifications were normalized to the housekeeping gene 18S.

### Phylogenetic analyses

All annotated R2R3 Myb domain-containing protein sequences were recovered by sequence similarity searches from the *Arabidopsis thaliana*, *Vitis vinifera*, *Brachypodium distachyon*, *Oryza sativa* (all from www.phytozome.net), and *Zea mays* B73 (www.maizegdb.org/) genome sequences. Protein sequences were aligned using Muscle (Edgar, 2004) and unambiguously aligned regions (102 amino acid sites) were selected with GBlocks (Castresana, 2000). The ProtTest software (Darriba, *et al.*, 2011) selected the JTT substitution model (Jones *et al.*, 1992) with four gamma distributed site rate categories as the best fit to the data. Initial distance analyses were performed using the programs Seqboot, Protdist, Neighbor and Consense from the PHYLIP package (Felsenstein, 1989). Subsequently, only sequences from the well-supported clade containing the *ZmMYB94* gene were realigned and 166 unambiguously alignable sites were selected for maximum likelihood bootstrap analysis using PHYML (Guindon *et al.*, 2010).

## Results

### The *fdl1-1* mutation causes abnormal seedling development

**Fig. 1.**
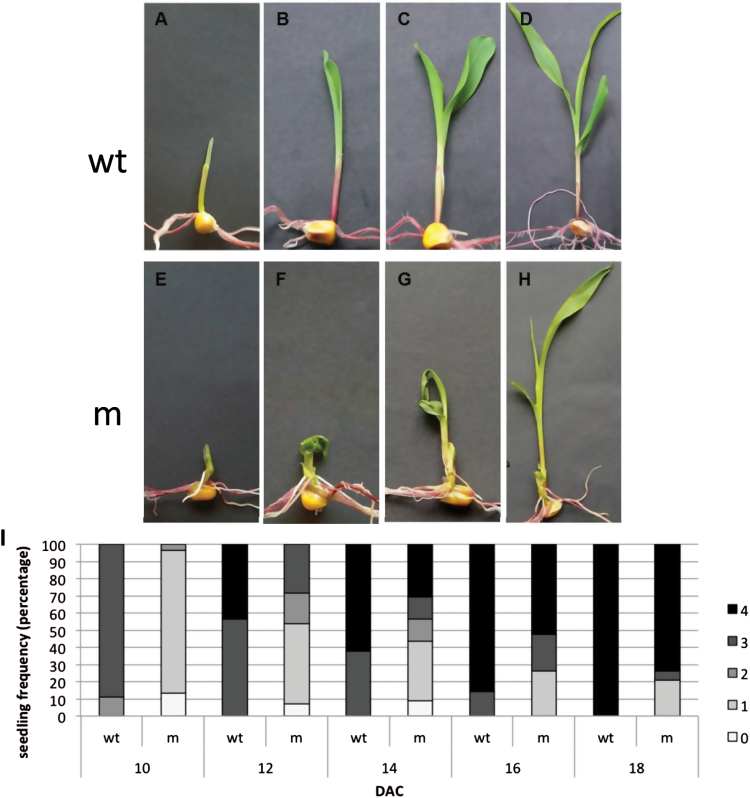
Seedling phenotypes and developmental profile of *fdl1-1*. Representative wild-type (A–D) and mutant (E–H) seedlings were photographed at successive stages of development: coleoptile (A, E), first leaf (B, F), second leaf (C, G) and third leaf (D, H). The developmental profile of fdl1-1 mutant (m) and wild-type (wt) seedlings is presented as percentage of seedlings in each stage at a specific time after initiation of cultures (I). Between 30 and 40 seedlings were analysed for each time point. 0, coleoptile presence; 1, first leaf presence; 2, second leaf presence, 3, third leaf presence; 4, fourth leaf presence; DAC, days after initiation of cultures.

The *fused leaves1-1 (fdl1-1*) recessive mutation, originally isolated in the F_2_ generation of a cross between a *En/Spm* line and a maize inbred line, was maintained by outcrossing *fdl1-1* homozygous plants to different inbred lines (A188, W64A, H99 and B73) for two or three generations. In all genetic backgrounds F2 progeny carrying the *fdl1-1* mutation segregated 3:1 for seedlings with abnormal morphology, which could be macroscopically distinguished from wild-type seedlings (Fig. 1).

The *fdl1-1* mutation specifically affected the early stages of seedling development, from germination to the 3–4 leaf stage, causing a general delay in germination and seedling growth as well as phenotypic abnormalities (Fig. 1E–G). An evident trait in mutant seedlings was the presence of a slightly thicker coleoptile (Fig. 1E). In contrast with wild-type leaves that elongated straight following their emergence from the coleoptile tip (Fig. 1A, B), mutant leaves emerged from the coleoptile in a warped manner, and appeared rolled and twisted (Fig. 1F, G). Moreover, regions of adhesion between the coleoptile and the first leaf, and between the first and the second leaf were observed and interfered with leaf expansion, causing bending of seedlings (Fig. 1G). After the third leaf stage, most of the mutant plants resumed normal phenotypic development and were indistinguishable from the wild-type plants (Fig. 1D, H).

The phenotypes of the fdl1-1 mutants were rather variable. The most severe phenotypes exhibited extremely stunted growth and heavily rolled (Supplementary Fig. S1A) or fractured (Supplementary Fig. S1B) leaves. In most of the mutant seedlings the first and second leaves appeared fused (Supplementary Fig. S1C) and showed remnants of the fusion at later stages (Supplementary Fig. S1D, E). To compare mutant and wild-type growth rate, seedlings belonging to the same F_2_ progeny were allowed to germinate on wet filter paper and, at succeeding time points, classified according to their stage of development into specific classes: presence of coleoptile (stage 0, Fig. 1A, E), first leaf (stage 1, Fig. 1 B, F), second leaf (stage 2, Fig. 1 C, G), third leaf (stage 3, Fig. 1D, H) and fourth leaf (stage 4). Ten days after culture initiation (DAC), the majority of wild-type plants had reached stage three, whereas the majority of the mutant seedlings were in stage one (Fig.1 I). Similarly, at later times (12 and 14 DAC), a substantial percentage of wild-type plants had already reached stage four, whereas mutant plants were at earlier stages. At 16 and 18 DAC, the discrepancy between wild-type and mutant was less consistent.

The strongest phenotypes, i.e. seedlings at stage one after 18 days of culture, representing 21.05 % of the mutant seedlings, arrested their growth, whereas the rest of the mutants, after the emergence of the third/fourth leaf, resumed almost normal growth, except for a reduction in their height. They could be grown to maturity and were fertile.

**Fig. 2.**
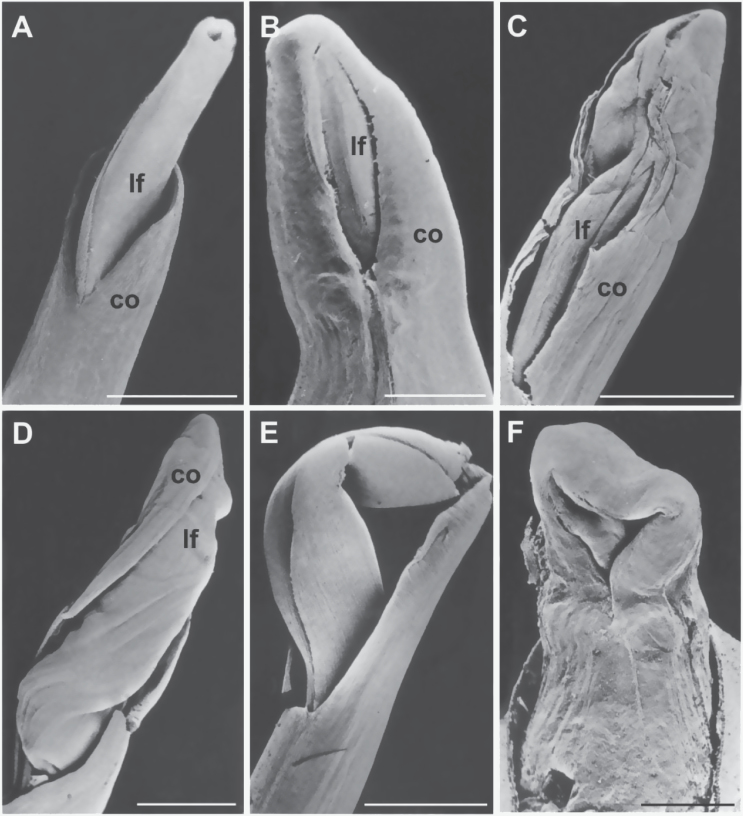
Scanning electron microscope micrographs of wild-type (A) and fdl1-1 mutant (B–F) germinating seedlings. (A) The first leaf emerges from the aperture at the top of a wild-type coleoptile. (B) A mutant coleoptile exhibiting a long subapical cleft. (C) A mutant coleoptile fractured by forcing leaves. (D) Leaves slipping laterally from the broken coleoptile; the leaf apical region is inside the closed top of the coleoptile. (E) An emerged leaf still joined with the coleoptile top. (F) A thick and greatly malformed mutant coleoptile. co, coleoptile; lf, leaf. Bars, 2mm.

To better examine the effect of the mutation on seedling organization, mutant germinating shoots were analysed by scanning electron microscopy. Mutant coleoptiles appeared thicker and variably misshapen (Fig. 2B–F) compared to wild-type (Fig. 2A). Soon after emergence wild-type coleoptiles exhibited an aperture at the top from which leaves easily emerged (Fig. 2A); in contrast, mutant coleoptiles often showed a lateral subapical slit (Fig. 2B). Mutant leaves did not emerge from the tip but from the side of the coleoptile by forcing and breaking its tissues (Fig. 2C, D), apparently because the upper leaf region was joined with the coleoptile apex (Fig. 2E). Finally, leaf detachment occurred through apical tissue tearing. In some greatly deformed mutant seedlings, coleoptile and first leaf were not easily distinguishable (Fig. 2F).

### The *fdl1-1* mutation affects embryonic shoot development and organ separation

To ascertain if the fdl1-1 phenotype was detectable prior to germination, both SEM and histological analysis were carried out on wild-type and homozygous mutant immature seeds from segregating F_2_ ears.

**Fig. 3.**
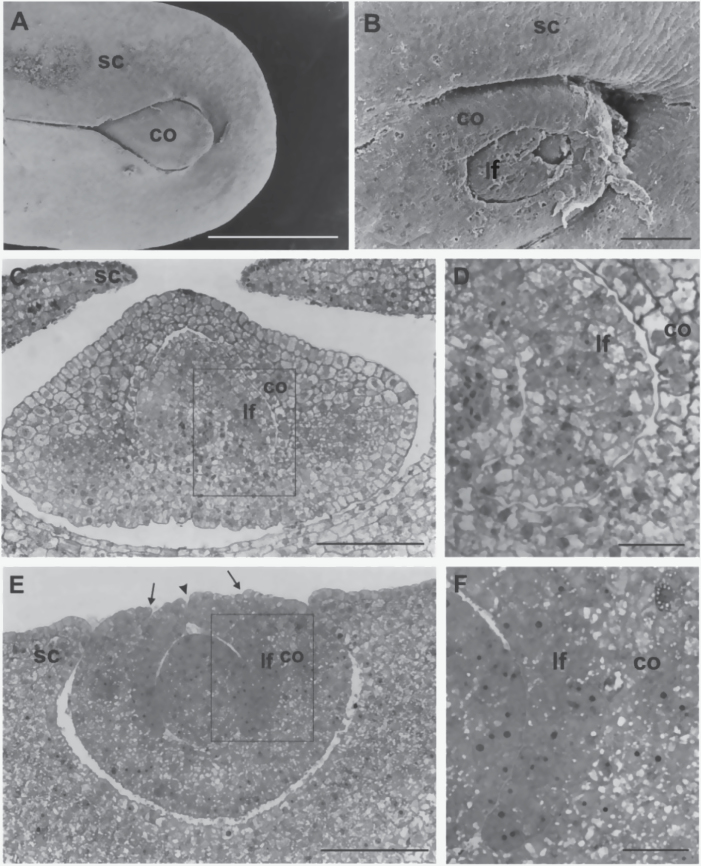
Scanning electron microscope (A, B) and light microscope (C–F) micrographs of the shoot region of 18 DAP embryos. (A) A wild-type embryo with the coleoptile enveloped by the scutellum. (B) A malformed fdl1-1 mutant embryo. Note the less curved scutellum, the uncovered shoot and the openings in coleoptile and first leaf surfaces. (C) The wild-type scutellum encloses the coleoptile, which contains the first leaf. (D) Higher magnification of the area framed in C shows the distinct coleoptile and leaf. (E) Laterally expanded scutellum, open coleoptile surface (arrows) and cleft leaf (arrowhead) are visible in the fdl1-1 mutant embryo shoot. (F) Higher magnification of the area framed in E. Note the lack of tissue identity between coleoptile and leaf in the fused region. co, coleoptile; lf, first leaf; sc, scutellum. Bars: 1mm (A); 100 μm (B, C, E); 25 μM (D, F).

SEM analysis revealed that in immature 18 DAP wild-type seeds (Fig. 3A) the embryo shoot was enveloped by the scutellum, from which only the apical region of the coleoptile emerged. By contrast, in mutant seeds (Fig. 3B) the scutellum is less curved, leaving the embryo shoot uncovered. Moreover, the embryo shoot exhibited external malformations with incomplete surfaces and irregular openings of coleoptile and first leaf. At the same stage light microscope observations of thin transversal sections disclosed the presence of wide areas of coleoptile and first leaf fusion in the mutant embryo (Fig. 3E, F) that were absent in wild-type embryos (Fig. 3C, D). In the fused regions the two organs were not clearly distinguishable, since attribution of cells to the coleoptile or leaf was not feasible (Fig. 3F). In addition, the sections showed that, contrary to wild type, the mutant scutellum did not extend towards the adaxial face of the embryo shoot and that the coleoptile and first leaf had incomplete and opened surfaces.

Taken together, these observations indicate that the effect of the *fdl1-1* mutation is exerted prior to germination. Accordingly, mutant seedlings cultivated through embryo rescue exhibited morphological defects. Seedlings showing all the typical fdl1-1 traits were detected, with the expected segregation ratio, in cultures obtained from immature embryos deriving from the same F_2_ ear (Supplementary Fig. S1F). Moreover, mutant seedling growth was reduced and delayed in comparison with that of wild-type siblings (Supplementary Fig. S1G). After 20 days of *in vitro* growth, most of the mutant seedlings (90%) from 12 DAP old embryos were at stage zero, whereas the majority of wild-type seedlings (80%) had already reached stage one. In cultures initiated from 18 DAP embryos the developmental profiles were less divergent. Among wild-type seedlings, stage two and three were the most represented (37.5% and 57.5% respectively); among mutant seedlings 55.2% reached stage three, while the growth of the remaining seedlings was at earlier stages.

### Analysis of tissues identities and cuticle and epicuticular wax deposition in mutant seedlings

**Fig. 4.**
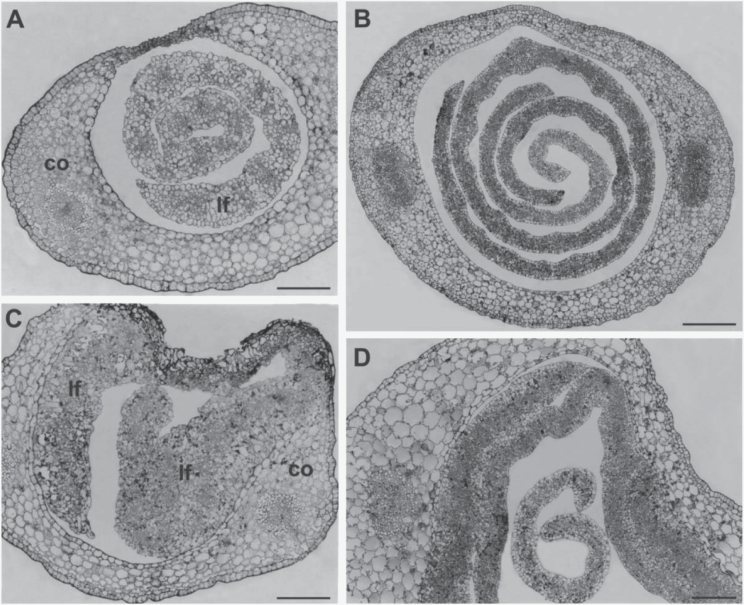
Light microscope micrographs of thin transversal sections of apical (A, C) and basal (B, D) regions of germinating seedling. (A) The apical region of a wild-type shoot shows a well-organized leaf in the hollow of the still closed coleoptile. (B) Young leaves independently enrolled are inside the coleoptile in the basal region of the wild-type shoot. (C) In the apical region of the mutant shoot masses of disorganized leaf tissues are fused with the coleoptile. (D) Fused areas between coleoptile and leaf surfaces are visible in the basal region of the mutant shoot. A young free leaf is also present. co, coleoptile, lf, leaf. Bars: 100 μm (A, C, D); 200 μm (B).

To better examine the effect of the mutation on the early phases of plant development, apical and basal cross-sections of wild-type (Fig. 4 A, B) and mutant (Fig. 4C, D) seedlings at the coleoptile stage were compared through light microscopy analysis. In the wild-type sections, leaves were enclosed and regularly rolled within the coleoptile and always appeared as distinct structures (Fig. 4A, B). In contrast, in the mutant apical sections, the inner surface of the coleoptile was fused with masses of a presumed first leaf tissue, lacking organization and exhibiting unclear cell identity (Fig. 4C). In the basal sections wide regions of fusion between coleoptile and first leaf epidermises and between epidermises of adjacent leaves were present (Fig. 4D).

**Fig. 5.**
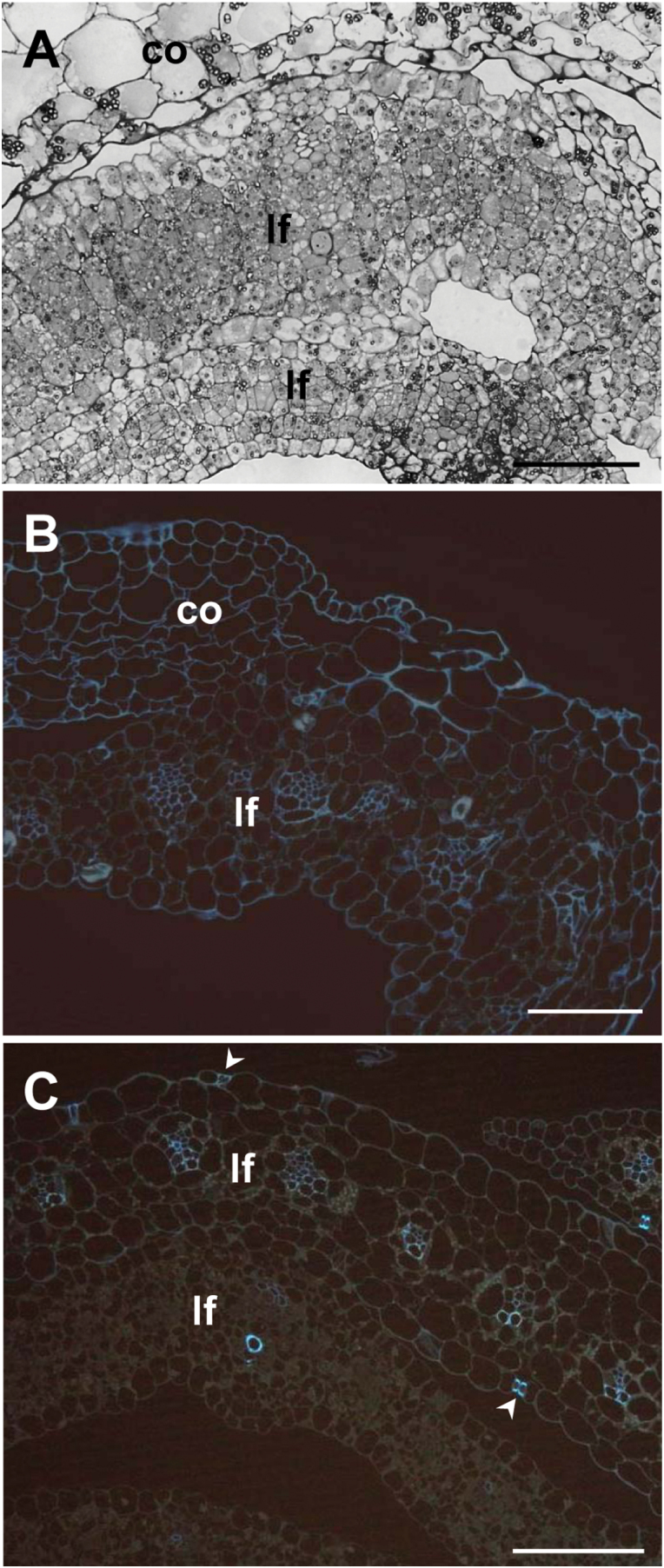
Light and fluorescence microscope micrographs of merged coleoptile and leaf blades in the fdl1-1 mutant. (A) At the light microscope some epidermal cell shapes are altered in areas involving coleoptile and first leaf fusion. Fused epidermal cells between two leaves look more regularly shaped. (B) At the fluorescence microscope severely misshaped cells can be seen in merged zone between undistinguishable leaf blade and edge of coleoptile lateral opening. (C) The tissue organization of fused blades from succeeding leaves does not show significant alterations. In the more differentiated leaf, xylem vessels are regularly distributed in the parallel bundles, the epidermises are recognizable and have stomata (arrow head) in the free regions. co, coleoptile; lf, leaf. Bars, 50 μm.

At higher magnification, areas of merged surfaces alternating with areas of free surfaces were visible (Fig. 5A). In areas involving coleoptile and first leaf fusion, epidermal cell shapes were frequently altered, whereas fused epidermal cells between two leaves assumed a more regular shape.

Fluorescence microscopy analysis of fused regions revealed that in the merged zone between leaf blade and edge of coleoptile lateral opening, cells of the two organs were indistinguishable and the boundary was no longer clearly recognizable (Fig. 5B). By contrast, the blades of adjacent leaves had recognizable epidermises with fused areas and free regions. The latter also showed well-differentiated stomata as evidenced by the intense autofluorescence of their cell walls (Fig 5C).

**Fig. 6.**
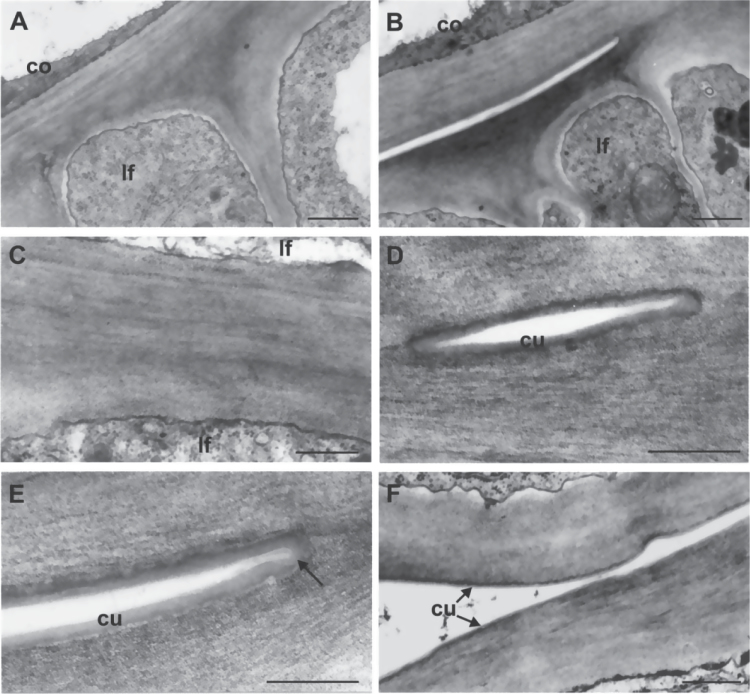
Transmission electron microscope micrographs of epidermal cell walls of coleoptile and first two leaves from the fdl1-1 mutant seedlings. (A) The cell wall proper of coleoptile and first leaf cannot be distinguished in a fused zone. (B) Distinct cell walls are visible in the free area adjacent to the fused one. (C) The higher magnification reveals the absence of any cuticular materials between the fused cell walls of two leaf surfaces. (D) A cuticle layer can be seen on the cell walls of the small free zone. (E) An evident continuous cuticle covers the free surfaces of the epidermal cells, also bordering the point of fusion (arrow). (F) The cuticle overlays the surfaces of two unfused leaf epidermises. co, coleoptile; cu, cuticle; lf, leaf. Bars: 1 μm (A, B); 0.5 μm (C, F); 0.25 μm (D, E).

TEM analysis revealed that, within zones of fusion between two organs, mutant cells were joined by an apparent single cell wall (Fig. 6A), the cell wall proper of each of them being undistinguishable. While a thick, continuous cuticle layer could be seen on the cell walls of separated epidermises in adjacent zones (Fig. 6B, D, F) covering the free surfaces of the two organs even in the initial point of separation (Fig. 6D, E), cuticular material was not detectable between joined cell wall zones even at higher magnification (Fig. 6C). Moreover, the cuticle was always detectable along free epidermises (Fig. 6F) and fusion was never found between cutinized surfaces of leaves or other plant organs.

Toluidine blue stained roots and seeds (which are devoid of cuticular material), whereas it did not stain aerial parts of wild-type or mutant seedlings, with the exception of mutant coleoptile fractured edges (Supplementary Fig. S2) since this dye permeates epidermal surfaces only if the cuticle is absent or defective. According to this assay (Tanaka *et al.*, 2004), the cuticle covering the free surfaces of mutant leaves displayed a waterproofing property.

**Fig. 7.**
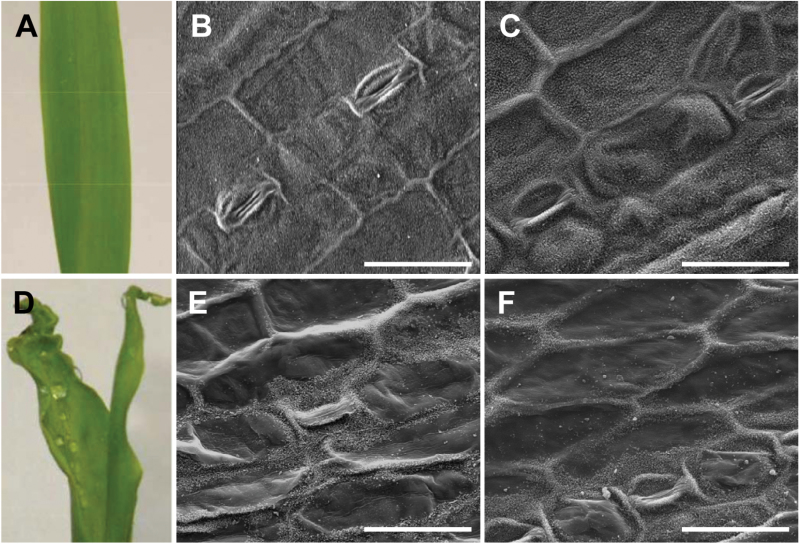
Second leaf of wild-type (A) and mutant (D) misted with water and micrographs of epicuticular waxes at scanning electron microscopy (SEM). The epicuticular waxes are regularly distributed on the upper surface of the second leaf of wild-type (B) while they show a patchy deposition on the same leaf of fdl1-1 (E). An irregular distribution of epicuticular waxes can be seen on the second leaf of an RNAi transformed seedling (F) if compared with the second leaf of an untransformed seedling (C). (This figure is available in colour at *JXB* online.)

It was also observed that, if mutant seedlings are misted with water, they retain water beads on their surface (Fig. 7D). SEM analysis suggested that this trait could be attributable to the alterations in epicuticular wax deposition, which was less homogenous on the mutant (Fig. 7E), than wild-type (Fig. 7B) surfaces. Indeed, mutant leaf surfaces exhibited a patchy distribution of epicuticular waxes, with some areas evenly covered and others completely devoid (Fig. 7E). This trait, similarly to the other fdl1-1 traits, was confined to the first two leaves, since later leaves show a regular distribution of epicuticular waxes that was indistinguishable from that of wild-type leaves (data not shown).

### The fdl1-1 mutant phenotype is linked to the insertion of a *En/Spm* element in a R2R3-MYB transcription factor

**Fig. 8.**
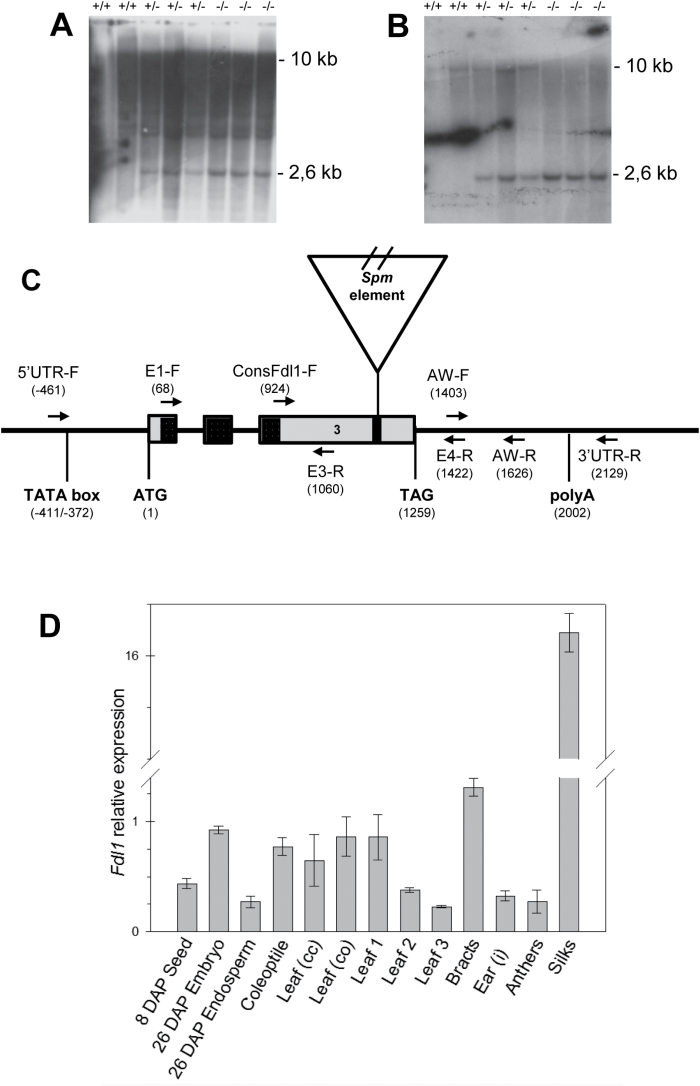
*ZmMYB94* gene structure and expression analysis. Southern analysis of *fdl1-1* and wild-type alleles (A, B). DNAs from homozygous wild-type (+/+), heterozygous (+/-) and homozygous mutant (-/-) seedlings were cleaved with BamH1. Filters were hybridized with the *En/Spm*-specific probe (A) or the 78bp fragment flanking the *En/Spm* insertion (B). (C) Schematic representation of the *ZmMYB94* gene showing the position of the three exons, indicated as grey rectangles, and the two introns, indicated as black lines. The *En/Spm* element inserted in the mutant allele is designated as a triangle, the 78bp genomic DNA flanking the insertion site, which was cloned after co-segregation analysis, is designed as a black rectangle and the region containing the MYB domain is highlighted in grey. The putative translational start (ATG) and stop (TAG) codons and polyA signal are reported. Exact positions are indicated with respect to the putative transcription initiation site. Names and positions of primers used for gene sequence analysis and qRT-PCR are also reported. The *En/Spm* element is not drawn to scale. (D) Relative expression level in different wild-type tissue samples from plants of the B73 inbred line. Expression level was established by qRT-PCR and the 18S gene was used as internal control. Bars represent means ±SD. DAP, days after pollination; cc, inside the closed coleoptile; co, emerging from the open coleoptile; i, immature. Tissue samples are listed in Supplementary Table S2.

Chromosomal arm location of *fdl1-1* was first established by means of TB-A translocations. Heterozygous +/*fdl1-1* females were crossed with a set of heterozygous or hyperploid B-A translocation males. Only the F_1_ obtained from crosses involving the TB-7L male parent uncovered the mutant phenotype, indicating that *fdl1* lies on the long arm of chromosome 7. A more refined position for *fdl1* was achieved through analysis of SSR marker distribution in a segregating F_2_ population obtained by crossing homozygous *fdl1-1/fdl1-1* females with H99 inbred male parents. Polymorphism for the markers UMC1125 and UMC1342 allowed the positioning of *fdl1* at 6.3 cM from the former and 5.4 cM from the latter. Since no similar mutants have so far been located in this genomic region, it was assumed that the fdl1 phenotype defines a novel gene. The fdl1-1 mutant had been isolated from an active *En*/*Spm* line, therefore a co-segregation analysis was performed to identify whether the insertion of an *En/Spm* element in the *fdl1* locus was causing the mutant phenotype. Genomic DNA was extracted from single individuals from F_1_ and F_2_ segregating progenies, including homozygous *fdl1-1/fdl1-1* mutants, *fdl1-1/+* heterozygous and *+/+* homozygous wild-types and compared by gel blot analysis. Hybridization of genomic DNAs with the *En*/*Spm*-specific probe pbx1, revealed a *BamH1* restriction fragment length polymorphism (RFLP) of approximately 2.6kb co-segregating with the mutant phenotype (Fig. 8A). Cloning of the RFLP highlighted the presence of 78bp of genomic sequence flanking the *En/Spm* element. When used as a probe against the *BamH1* blot, this fragment identified the same 2.6kb mutant RFLP in *fdl1-1/+* and *fdl1-1*/*fdl1-1* individuals (Fig. 8B) as well as a 10kb restriction fragment ascribable to the wild-type allele of *fdl1-1/+* and *+/+* individuals (Fig. 8B). Co-segregation analysis, performed with both the *En/Spm* probe and the genomic probe on a total of 50 individuals (data not shown) indicated that the element was inserted either in the *fdl1* gene itself or in a closely linked locus. Alignment of the 78bp flanking sequence with the maize genome sequence (www.maizesequence.org) placed the *En/Spm* insertion in the GRMZM2G056407 gene model corresponding to the *ZmMYB94* gene, which is located on chromosome 7L in the region delimited by the UMC1125 and UMC1342 markers where the *fdl1* gene was mapped.

To experimentally validate the gene model, overlapping amplification products were obtained with gene specific primer sets reported in Fig. 8C (5′UTR-F and E3-R, ConsFdl1 and 3′UTR-R), from wild-type genomic DNA and cDNA and sequenced. Sequences were assembled to generate a contig showing 100% identity with the GRMZM2G056407 gene prediction comprising three exons separated by two introns (Fig. 8C), whose putative product consists of 330 amino acids. Sequence analysis of mutant DNA confirmed that the *En/Spm* element insertion was within the GRMZM2G056407 coding sequence (data not shown).

To verify the results obtained from the Southern-based co-segregation analysis, a PCR-based analysis was performed on 100 mutant and 40 wild-type F_2_ individuals, whose genotypes were ascertained by selfing. The two products, of 309bp and 625bp respectively, which are specific for the mutant allele (Supplementary Fig. S3C, D), were always and exclusively present in all mutant samples, whereas the third amplification product of 692bp, was only yielded by wild-types (Supplementary Fig. S3B). No recombination was detected between the *fdl1-1* allele and the *En/Spm* insertion in the *ZmMYB94* gene, indicating a tight linkage between this insertion and the *fdl1-1* mutation.

Mutant cDNAs were also amplified by using the same primer sets (Consfdl2-F, Spm1-R; and Spm3-F, AW-R) and produced amplicons of the same size as those obtained from genomic DNA (data not shown). Sequence analysis showed that the *fdl1-1* mutant transcript retains the *En*/*Spm* element and is predicted to encode a non-functional protein carrying a frameshift mutation at amino acid position 278 and a premature stop codon at position 298.

Seed progeny obtained from crossing *fdl1-1* with a waxy (wx) endosperm line homozygous for the *wx-m8* allele (Shure *et al.*, 1983), which contains a defective *En/Spm* element, did not highlight any Waxy revertant sector, suggesting that the element inserted in the *fdl1* locus is a non-autonomous element. A similar conclusion arose from the PCR based analysis of DNA extracted from homozygous entire mutant leaves fractionated in sectors. The primer set specific for the mutant allele, i.e. ConsFdl2-F and Spm1-R, gave the expected product, whereas the primer set specific for the wild-type allele, i.e. ConsFdl2-F and AW-R, did not produce an amplicon (data not shown).

Attempts to generate germinal revertants by crossing the mutant line with a line containing an autonomous *En/Spm* element, as well as to isolate novel transposon-induced alleles through *Mutator*-targeted mutagenesis were unfruitful (data not shown). Proof of gene identity was thus obtained using an alternative approach consisting of an RNAi experiment, in which *ZmMYB94* as well as the closely related paralogue *ZmMYB70* (GRMZM2G139284), were targeted. Six independent transformants were obtained and T_1_ progenies, which showed 50% segregation of transgenic individuals, as expected for single locus insertion, were visually scored for mutant phenotypes. Although the mutant phenotype was very mild and rather sensitive to environmental conditions, transgenic seedlings of two transformants grown at 23°C showed some typical fdl1-1 mutant traits consisting of curly leaves and regions of adhesions between the first two leaves (Supplementary Fig. S4B, C, D). In these two transformants the relative expression level of *ZmMYB94* in the third leaf was 4% and 5% respectively of that observed in wild-type.

Moreover, SEM analysis of a transformed mutant second leaf revealed an uneven epicuticular wax distribution on its epidermal surface (Fig. 7F) resembling that of the fdl1-1 mutant epidermal surface (Fig. 7E). This pattern was not detected on the second leaf of an untransformed sibling plant (Fig.7C).

### The expression profile of the *ZmMYB94* gene in the seedling overlaps with the pattern of mutant phenotype expression

The spatial and temporal expression profile of the *ZmMYB94* gene was analysed by qRT-PCR in different maize tissues (Fig. 8D; Supplementary Table S2), using the AW-F and AW-R *ZmMYB94* specific primers. The *ZmMYB94* mRNA was detected in all organ/tissues analysed, except in the primary root, and the expression pattern obtained (Fig. 8D) did not deviate from that described in the work of Sekhon *et al.*, (2013). The highest expression (5-fold higher than other tissues) was observed in ear silks. Within the seed, *ZmMYB94* was more expressed in the embryo than the endosperm. In the seedling, higher expression levels were observed in the coleoptile and in the first leaf, with a progressive decrease of expression in the second and third leaf. We note that in seedlings, the expression profile detected by qRT-PCR analysis matches the pattern of fdl1-1 phenotypic expression observed (Fig. 1).

### Phylogenetic analysis

**Fig. 9:**
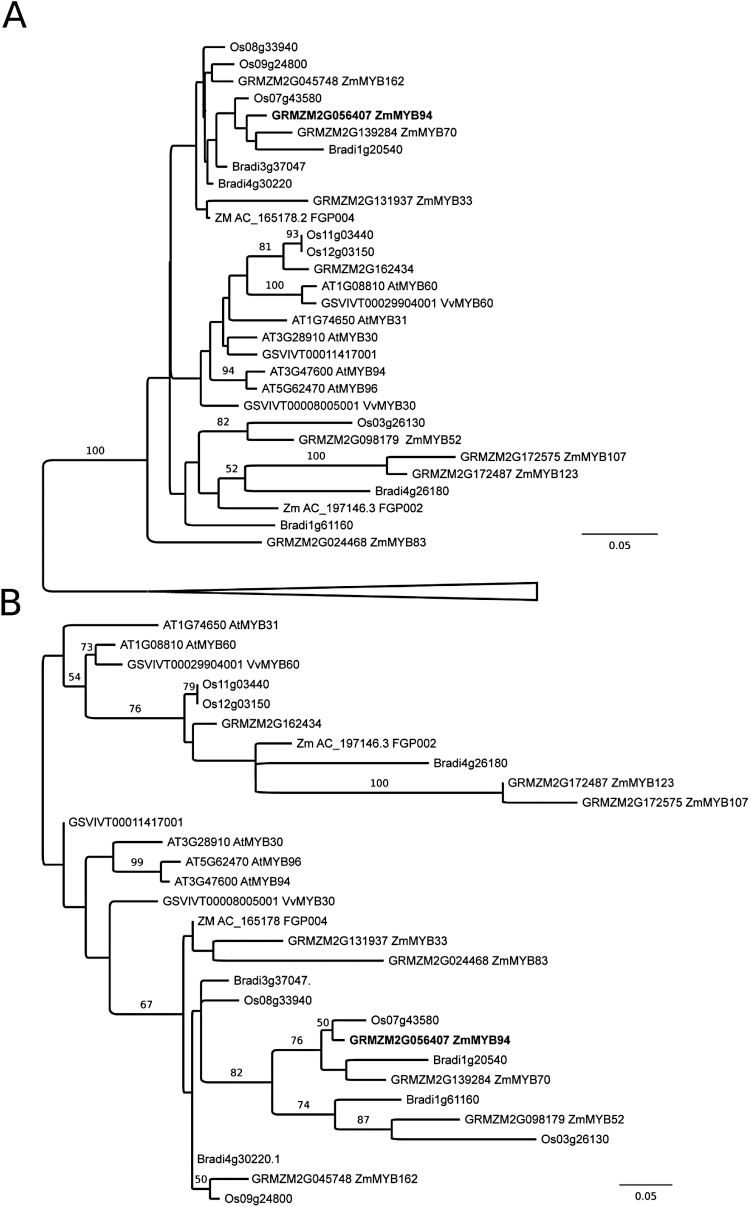
Phylogenetic placement of *ZmMYB94*. (A) Distance tree of all annotated R2R3-MYB protein sequences from *Zea mays*, *Oryza sativa*, *Brachypodium distachyon*, *Arabidopsis thaliana* and *Vitis vinifera* (104 unambiguously aligned amino acid positions). The tree is arbitrarily rooted at the base of the well-supported clade that contains ZmMYB94 and all relationships outside this clade collapsed. (B) Arbitrarily rooted maximum likelihood phylogenetic reconstruction of relationships within the ZmMYB94 clade (166 amino acid positions) from the same taxonomic sampling employed in panel A. For both panels, bootstrap support values for internal branches are marked where greater than 50%.

Preliminary phylogenetic reconstructions considering 104 unambiguously alignable amino acid residues from the highly conserved MYB domains of all annotated R2R3-MYB proteins from *Z. mays*, *O. sativa*, *B. distachyon*, *A. thaliana* and *V. vinifera* indicate that *ZmMYB94* falls within a well supported clade containing representatives of all species considered (Fig. 9A), but did not allow confident assignment of an exact phylogenetic placement. Consideration of only sequences within this clade allowed a subsequent phylogenetic reconstruction including 166 aligned amino acid sites (Fig. 9B), which, while failing to unambiguously resolve all relationships within the MYB subclade, provided strong support for co-orthology of *ZmMyb94* and *ZmMyb70* with functionally uncharacterized *Brachypodium* and rice genes and as part of a monocot-specific gene family expansion. Our analyses recover no support for direct orthology between *ZmMyb94* or *ZmMyb70* and any dicot homologue. Indeed, the most closely related dicot MYBs were *V. vinifera MYB30*, an additional uncharacterized *V. vinifera* MYB and a clade of recently duplicated *Arabidopsis* MYBs (*AtMYB30/94/96*). The absence of direct dicot orthologues of *ZmMyb94* notwithstanding, it is interesting to note that *MYB30*, *MYB94* and *MYB96* have all been implicated in the regulation of cuticular wax biosynthesis in *Arabidopsis* (Shepherd *et al.*, 2006; Raffaele *et al.*, 2008; Seo *et al.*, 2011; Lee *et al.*, 2014).

## Discussion

Seed germination and seedling emergence represent crucial events in the plant life cycle, and rapid and robust emergence allows plants to take advantage of favourable environments and to compete with neighbours. Moreover synchrony in seedling emergence is a profitable character for cultivated plants because it facilitates the weed control practices (Buhler *et al.*, 2000). The study of the *fd11*-1 mutant carried out in this work adds knowledge on the molecular mechanisms underlying shoot formation during embryogenesis, germination and early seedling development in *Z. mays*. The fdl1-1 seedling phenotype is characterized by curly leaves with regions of adhesion between the first leaf and the coleoptile or between the first and the second leaves. In these regions suture zones devoid of any intervening cuticular material between juxtaposed cell walls are present (Fig. 6), which are adjacent to free zones, where cell wall surfaces are covered by a continuous cuticle layer. Indeed, while most of the young epidermis is covered by a well-organized cuticle making them waterproof (Supplementary Fig. S2), an additional feature of the mutant seedling is a patchy distribution of epicuticular waxes on the epidermis of the second leaf, where areas devoid of epicuticular waxes are clearly visible (Fig. 7E).

The *fdl1-1* mutant, located on the long arm of chromosome seven, has been ascribed to the insertion of an *En/Spm* transposable element inside the third exon of a maize R2R3-MYB transcription factor whose gene model has been named either *ZmMYB94* in the last version of the maize genome sequence (http://www.maizesequence.org) or *ZmMYB110* in the paper of Du *et al.*, (2013). The decision to adopt the first designation in this work is based on the relationship, mentioned hereafter, with *AtMYB94.*

*ZmMyb94,* and its close paralogue *ZmMyb70,* are members of a monocot-specific local expansion of the MYB family, within which *ZmMyb94* is the first member to be functionally analysed. The function of *ZmMYB94* recalls that of *AtMYB96*, *AtMYB94* and *AtMYB30,* which are its closest *Arabidopsis* members. *AtMYB96* is involved in the activation of cuticular wax biosynthesis (Seo *et al.*, 2011). *AtMYB30* acts as a transcriptional activator of genes encoding the fatty acid elongase complex, being responsible for the biosynthesis of very long-chain fatty acids (VLCFA), which are leaf epidermal wax components (Shepherd *et al.*, 2006; Raffaele *et al.*, 2008). *AtMYB94* has recently been implicated in regulation of wax deposition through direct binding of promoters of various genes involved in wax biosynthesis (Lee *et al.*, 2014). The action of these *Arabidopsis* genes has been correlated with environmental responses (Shepherd *et al.*, 2006; Raffaele *et al.*, 2008; Seo *et al.*, 2011; Lee *et al.*, 2014) but not, to our knowledge, with developmental processes.

The relationship between alterations in epidermis covering material and developmental defects, in particular organ fusion, has been reported (Nawrath, 2006; Bernard and Joubès, 2013). For example, the maize *adherent1* mutant exhibits defective cell wall and epicuticular wax deposition and shows organ fusion (Sinha and Linch, 1998). In *Arabidopsis*, organ fusions were observed in mutants such as *fiddlehead-1* (Lolle *et al*., 1992, 1997; Yephremov *et al.*, 1999; Pruitt *et al.*, 2000), *bodyguard* (Kurdyukov *et al.*, 2006a) and *hothead* (Kurdyukov *et al.*, 2006b), which are defective in the biosynthesis of cuticle components as well as in the *dso* (*DESPERADO*) mutant, defective in a transporter required for cutin and wax secretion (Panikashvili *et al.*, 2007). Postgenital fusion also occurred in the *lacerata* mutant of *Arabidopsis* in which cutine biosynthesis is impaired (Wellesen *et al.*, 2001), and in the *cer3*/*wax2* mutant, specifically impaired in the elongation of very long-chain (VLC) alkanes, which are major components of cuticular waxes (Chen *et al.*, 2003; Rowland *et al.*, 2007). Organ fusion has also been observed in the *lacs1/lacs2* double mutant whose corresponding genes are involved in cuticular wax and cutin biosynthetic pathways, respectively (Weng *et al.*, 2010).

Particular analogies can be recognised between *fdl1-1* and *bodyguard* (Kurdyukov *et al.*, 2006a) as both exhibit direct adhesion of cell walls in the suture zones that are devoid of intervening cuticular membrane (Fig. 6), and between *fdl1-1* and *lacerata,* the latter forming suture zones in which the cuticle was not particularly conspicuous, and wherein contacting cell walls adhere directly to each other (Wellesen *et al.*, 2001). Our functional study of the *fdl1-1* mutant provides further confirmation that an intact cuticle layer is necessary for establishing organ boundaries by preventing post-genital organ fusion and is consistent with analyses of *Arabidopsis* plants expressing a fungal cutinase and exhibiting leaf fusions, in which cell walls of both epidermal cells came into direct contact (Sieber *et al.*, 2000).

Additional developmental defects in the fdl1-1 mutant include delays in germination and seedling elongation (Fig. 1) as well as the presence of a thicker coleoptile, with impaired opening (Fig. 2). Mutant leaves are forced to start growing when still within the coleoptile and instead of emerging through a clear-cut hole at the apex of the coleoptile, emerge from the lateral side of this organ (Figs 1, 2). Fdl1-1 mutant seedling defects can be traced back to abnormalities in the embryogenetic process. At 18 DAP (Fig. 3E), as well as at later developmental stages (data not shown), both scutellum and coleoptile are not properly developed in correspondence with the adaxial side of the mutant embryo. Leaf primordia are abnormal and not regularly rolled and coleoptile and first leaf primordia are fused (Fig. 3F). It is thus conceivable that the *fdl1* gene begins to exert its influence when coleoptile and first leaf primordia are initiated.

Seedling phenotypes display variability in phenotypic expression at early stages of seedling growth (Supplementary Fig. S1). However after stage three (presence of third leaf), most homozygous individuals, bearing either mild or severe phenotypes, resumed a wild-type phenotype (Fig. 1). This observation, together with the mRNA expression profile (Fig. 8D), suggests that *fdl1* gene action is exerted during a strict developmental window. In maize, juvenile and adult leaves differ since the former are coated with epicuticular waxes and lack specialized cells, whereas the latter lack epicuticular waxes, bear trichomes and bulliform cells and have crenulated epidermal cell walls (Sylvester *et al.*, 2001). There is evidence that the juvenile and adult phases are genetically separated. The action of *glossy15*, an *APETALA2*-like gene, is required to maintain juvenile leaf identity and its down-regulation promotes phase change (Moose and Sisco, 1996; Lauter *et al.*, 2005). The glossy appearance of the first fdl1-1 leaves and the stronger expression of *ZmMYB94* in the first leaves are indications that *ZmMYB94* may be another actor in phase change and it would be interesting to investigate regulatory links between *ZmMYB94* and *Gl15*.

In conclusion, *ZmMYB94*, whose action is specific for embryogenesis and early phase of seedling development, is required for determining a regular pattern of cuticle deposition in the young shoot, which is shown to be a prerequisite for the establishment of organ boundaries and identities. As outlined in the study of the maize OUTER CELL LAYER1 (OCL1) gene, cuticle deposition implies the coordinated activity of different regulatory factors (Javelle *et al.*, 2011). Further analysis of the MYB94 action will contribute to the understanding of this regulatory network.

## Supplementary data

Supplementary data are available at *JXB* online.

Supplementary Table S1. List of primers used in this study.

Supplementary Table S2. List of tissues used for qRT-PCR.

Supplementary Figure S1. Variable fdl1-1 seedling phenotypes.

Supplementary Figure S2. Response of wild-type and fdl1-1 mutant seedlings to the toluidine blue test.

Supplementary Figure S3. PCR based co-segregation analysis.

Supplementary Figure S4. Phenotype of RNAi transformed seedlings.

Supplementary Data
