# Supplementary material for: The maize fused leaves1 (fdl1) gene controls organ separation in the embryo and seedling shoot and promotes coleoptile opening
Source: J Exp Bot. 2015 Jun 20;66(19):5753–67. doi: 10.1093/jxb/erv278 (PMC4566974; doi:10.1093/jxb/erv278)
Supplement: Supplementary Data [file supp_66_19_5753__index.html]

The maize fused leaves1 (fdl1) gene controls organ separation in the embryo and seedling shoot and promotes coleoptile opening — The maize fused leaves1 (fdl1) gene controls organ separation in the embryo and seedling shoot and promotes coleoptile opening — Supplementary Data 

# The maize *fused leaves1* (*fdl1*) gene controls organ separation in the embryo and seedling shoot and promotes coleoptile opening

## Supplementary Data

Data files

- Supplementary Data - Supplementary Data
