# Supplementary material for: The maize fused leaves1 (fdl1) gene controls organ separation in the embryo and seedling shoot and promotes coleoptile opening
Source: J Exp Bot. 2015 Jun 20;66(19):5753–67. doi: 10.1093/jxb/erv278 (PMC4566974; doi:10.1093/jxb/erv278)
Supplement: Supplementary Data [file supp_erv278_jexbot143990_file001.pdf]

The maize *Fused leaves1* (*Fdl1*) gene controls organ separation in the embryo and seedling shoot and promotes coleoptile opening

Nicoletta La Rocca, Priscilla Manzotti, Marina Cavaiuolo, Alessandra Barbante, Francesca Dalla Vecchia, Damiano Gabotti, Ghislaine Gendrot, David S. Horner, Jelena Krstajic, Martina Persico, Nicoletta Rascio, Peter Rogowsky, Alessio Scarafoni and Gabriella Consonni

Table S1. List of primers used in this study.

A) Primers for genomic and cDNA cloning and sequence analysis

**Gene**	**Primer name**	**Sequence**	**T**
ZmMYB94	5'UTR-F	5'-GAGCACAGCACAAAAGGTCA-3'	58
	E1-F	5'-CGTCCTCGTCTCCTACATCC-3'	54
	ConsFdl1-F	5'-CAAGAACTACTGGAACACGC-3'	56
	E3-R	5'-CCAGGTTGATGTCCGTCTG-3'	58
	E4-R	5'-GCATCCGCGAGATGTTCT-3'	54
	3'UTR-R	5'-TAAAATCCTGAGCCCTTGGA-3'	58

B) Primers for co-segregation analysis

**Gene**	**Primer name**	**Sequence**	**T**
ZmMYB94	ConsFdl2-F	5'-GAGAACATCTCGCGGATGC-3'	58
	AW-R	5'-CCACACAACATGCAACTTGC-3'	60
En/Spm	Spm1-R	5'-CGTCGGTTTCATCGGGACC-3'	58
	Spm3-F	5'-TGACGGCTAAGAGTGTCGG-3'	58

C) Primers for qRT-PCR analysis

Gene	Primer name	Sequence	T
ZmMYB94	AW-F	5’-TAGCTGTTCAGATCGGTCG-3’	60
	AW-R	5’-CCACACAACATGCAACTTGC-3’	60
rRNA18S	18S -F	5’-GGAGCCATCCCTCCGTAGTTAGCTTCTT-3’	68
	18S R	5’-CCTGTCGGCCAAGGCTATATACTCGTTG-3’	68

D) Primers for RNAi cloning

Gene	Primer name	Sequence
ZmMYB94	attB1-FDL-RNAi	5'-CAAGTTTGTACAAAAAAGCAGGCTCTCGACGACCTCAAGCCGCAGCTGCAG-3'
	attB2-FDL-RNA-	5'-CCACTTTGTACAAGAAAGCTGGGTTGGCGCTCTGGACAGCATGGTG-3'

T: annealing temperature for PCR expressed in °C

Table S2. List of tissues used in this study for qRT-PCR

**Sample name**	**Age**	**Tissue description**
Kernel 8	8 DAP	Whole kernels
Embryo 26	26 DAP	Isolated embryos
Endosperm 26	26 DAP	Isolated endosperms
Coleoptile	4 DAC	Closed coleoptiles
Coleoptile leaf (cc)	6 DAC	Leaves enclosed in closed coleoptiles
Coleoptile leaf (co)	8 DAC	Leaves enclosed in open coleoptiles
Leaf 1	9 DAC	Developing first leaves collected at the emergence of the second leaf
Leaf 2	10 DAC	Developing second leaves collected at the emergence of the third leaf
Leaf 3	12 DAC	Developing third leaves collected at the emergence of fourth leaf
Primary root	9 DAC	Primary seedling roots dissected at the first leaf stage
Ear	n.d.	Immature ears
Bracts	n.d.	Bracts removed from immature ears
Silk	n.d.	Mature silks
Anther	n.d.	Whole anthers dissected from tassel, including pollen

DAP: days after pollination; DAC: days after initiation of cultures; n.d. not determined

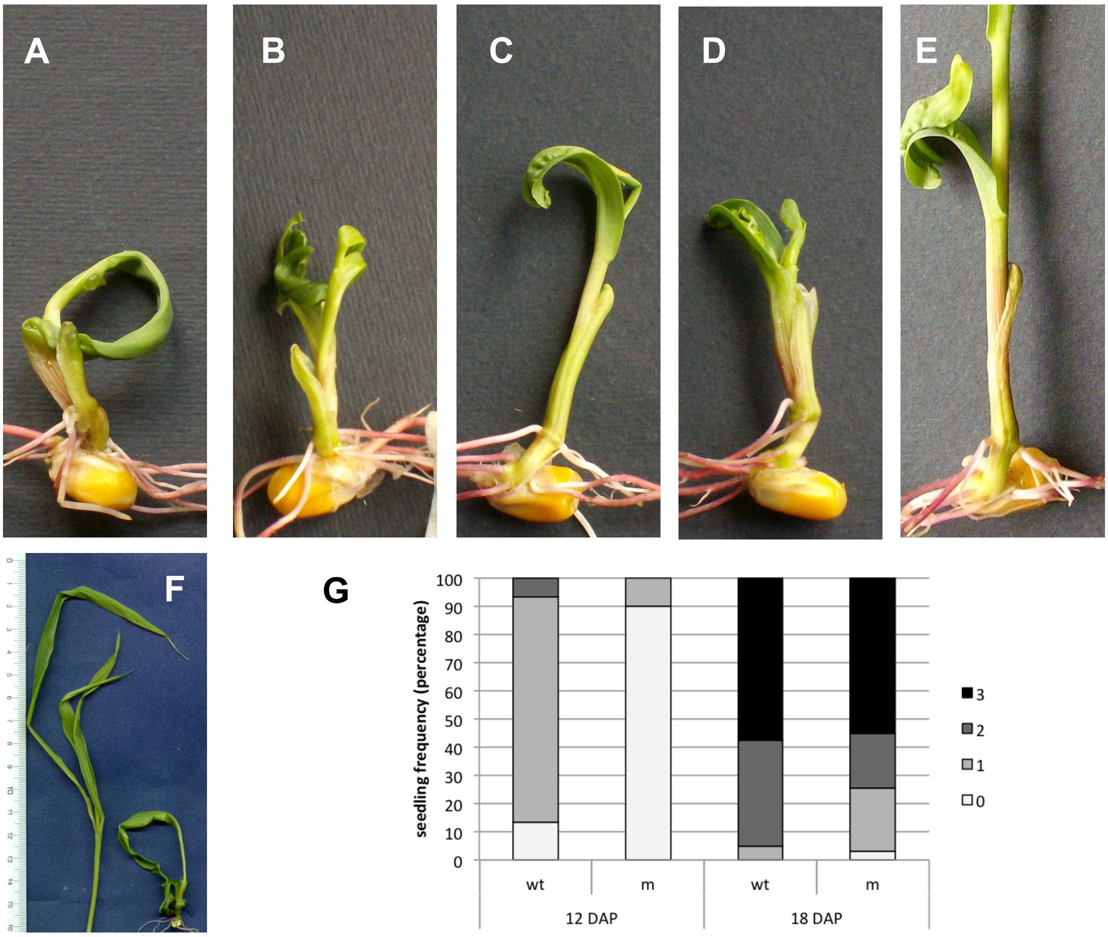

Supplementary Figure S1. Variable fdl1-1 seedling phenotypes. Mutant seedlings with extremely stunted growth and heavily rolled (A) or fractured leaves (B), with fusion occurring between first and the second leaf (C) and showing remnants of the fusion at later stages (D, E). (F) Representative wild-type (left) and mutant (right) seedlings obtained from cultivated immature embryos. (G) The developmental profile of fdl1-1 mutant (m) and wild-type (wt) seedlings is presented as percentage of seedlings in each stage of development. Mutant embryos were isolated at 12 and 18 days after pollination (DAP). Between 30 and 40 seedlings were analyzed for each time. (0: coleoptile presence, 1: leaves emerging and first leaf presence, 2: second leaf presence, 3: third leaf presence, 4: fourth leaf presence).

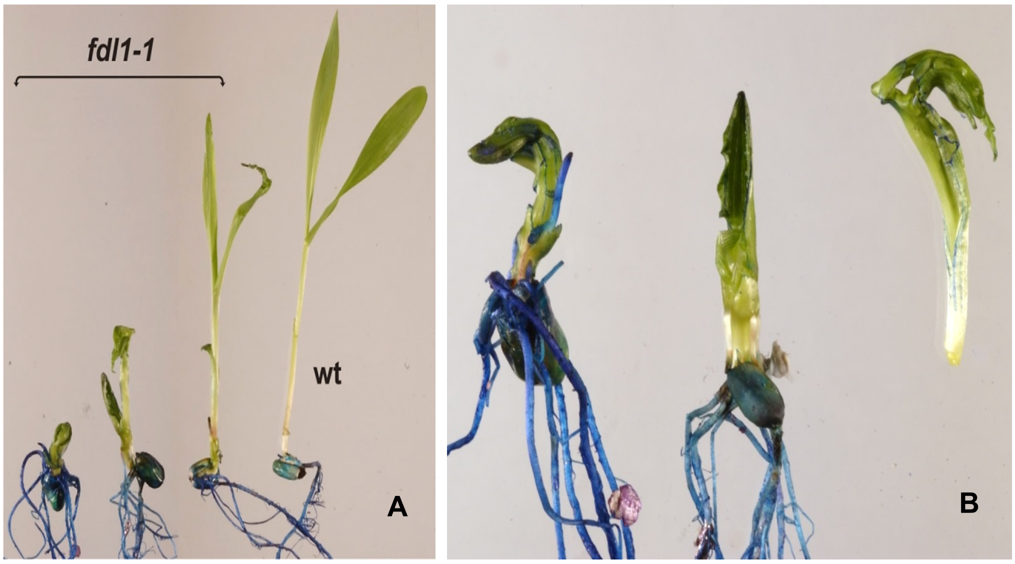

Supplementary Figure S2. Response of wild-type and fdl1-1 mutant seedlings to the toluidine blue test. (A) After three min of submersion of seedlings in the toluidine blue solution, all the roots and kernel integuments appeared stained, whereas the aerial organ surfaces remained unstained in both mutant and wild-type seedlings, with the exception of the fractured edges of the fdl1-1 coleoptile. (B) Higher magnifications show the staining only at the fractured edges of the fdl1-1 coleoptiles.

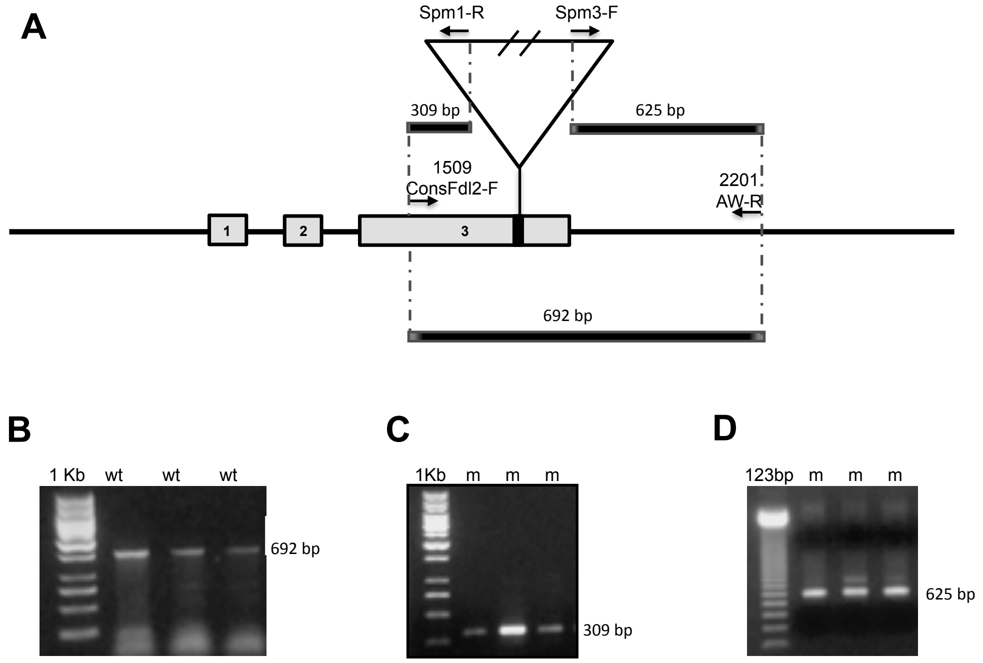

Supplementary Figure S3. PCR based co-segregation analysis. (A) Schematic representation of the *fdl1* gene with indicated the ConsFdl2/AW-R primer set, specific for the wild-type allele, the ConsFdl2-F/Spm1-R and Spm3-F/AW-R primer sets, specific for the mutant allele, used in the analysis.

PCR products from the wild-type (B) and mutant alleles (C, D). (wt: wild-type; m: mutant; 123 bp: 123 bp DNA ladder; 1kb: 1 kb DNA ladder)

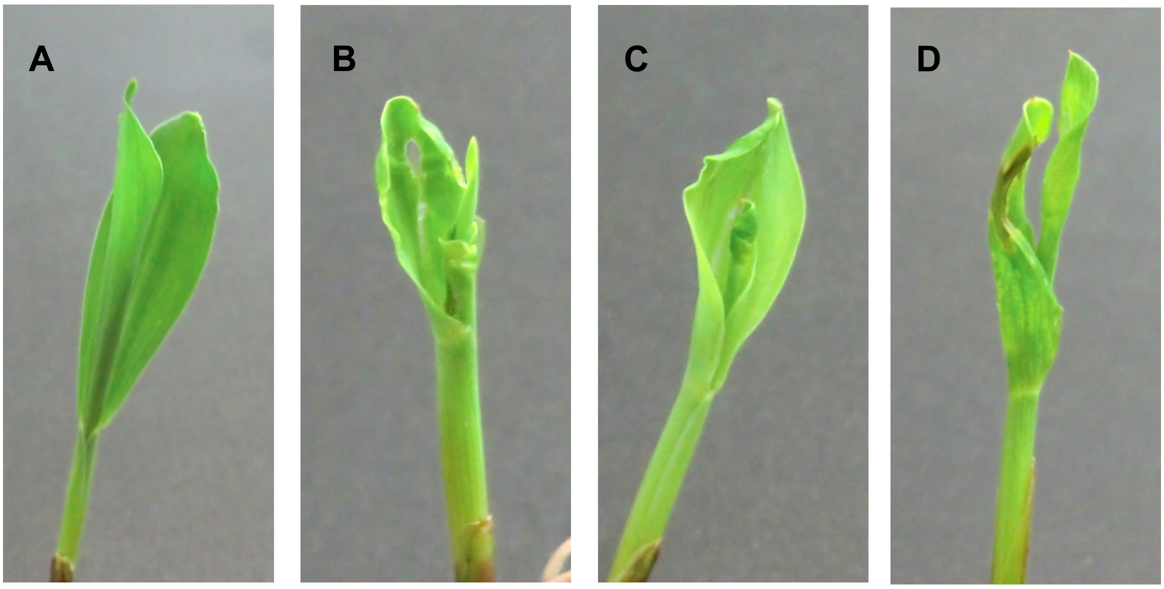

Supplementary Figure S4 Phenotype of RNAi transformed seedlings. Untransformed (A) and RNAi transformed (B,C,D) phenotypes showing curly leaves. The split on the first leaf (B) indicates a former adhesion with a different leaf when enrolled inside the coleoptile.
